# What can areas of low *Dirofilaria immitis* preventive governance teach us?

**DOI:** 10.1590/S1984-29612026037

**Published:** 2026-08-03

**Authors:** Norma Labarthe, Simone Cristina Rosa, Flavya Mendes-de-Almeida, Fabiana Knakfuss, Roberto Teixeira, Bruno Alberigi, João Palmer, Camila Class, Lucas Lobão, Alynne da Silva Barbosa

**Affiliations:** 1 Universidade Federal Rural do Rio de Janeiro – UFRRJ, Programa de Pós-graduação em Ciências Veterinárias, Seropédica, RJ, Brasil; 2 MSD Saúde Animal, São Paulo, SP, Brasil; 3 Universidade Federal Fluminense – UFF, Departamento de Patologia e Clínica Veterinária, Niterói, RJ, Brasil; 4 Afya Universidade Unigranrio, Curso de Medicina Veterinária, Duque de Caxias, RJ, Brasil; 5 UNIVET Clínica Veterinária, Rio de Janeiro, RJ, Brasil; 6 Universidade Federal Rural do Rio de Janeiro – UFRRJ, Departamento de Medicina e Cirurgia Veterinária, Seropédica, RJ, Brasil; 7 Universidade Federal Fluminense – UFF, Departamento de Microbiologia e Parasitologia, Niterói, RJ, Brasil

**Keywords:** Dirofilaria immitis, heartworm, chemoprophylaxis, preventive governance, risk factors, Dirofilaria immitis, dirofilariose, quimioprofilaxia, governança preventiva, fatores de risco

## Abstract

*Dirofilaria immitis* remains a significant canine pathogen in Brazil despite the long-standing availability of chemoprophylactic products. Higher infection rates in low-income areas, and the persistence of cases in populations with veterinary access, suggest gaps in preventive governance. A cross-sectional survey was conducted in two high-challenge areas of metropolitan Rio de Janeiro, Brazil, from June 2023 to January 2024. Dogs older than 1 year and not on heartworm prophylaxis during the previous 6 months were enrolled. Blood samples were tested for *D. immitis* antigen, and owner questionnaires captured demographic, environmental and clinical data. Risk factors were evaluated using binomial regression models. Among 526 dogs sampled, the overall antigen-positive rate was 22.6%, with a higher prevalence in Niterói-Maricá (27.0%) than in the Western area of the Rio de Janeiro municipality (18.15%). Male, short-coated, heavier or outdoor-living dogs had significantly higher odds of infection. Coughing was more frequent in antigen-positive dogs, while no significant associations with weight loss or lethargy were observed. Despite widespread veterinary recommendations, substantial infection rates persist, especially in low-income communities, underscoring failures in preventive governance. Integrated efforts from veterinarians, regulatory authorities and the animal-health industry are urgently needed to improve chemoprophylaxis uptake and protect canine health in endemic regions.

## Resumo

*Dirofilaria immitis* continua sendo um patógeno canino relevante no Brasil, apesar da disponibilidade de quimioprofiláticos há três décadas. Taxas elevadas em áreas de baixa renda e a persistência de casos em populações com acesso veterinário sugerem falhas na governança preventiva. Inquérito transversal foi conduzido em duas áreas de alto desafio na região metropolitana do Rio de Janeiro, Brasil, entre junho de 2023 e janeiro de 2024. Incluíram-se cães acima de 1 ano sem quimioprofilaxia nos seis meses anteriores. Amostras sanguíneas foram testadas para antígeno de *D. immitis* e questionários coletaram dados demográficos, ambientais e clínicos. Fatores de risco foram avaliados por regressão binomial. Entre 526 cães, a positividade global foi de 22,6%, maior em Niterói-Maricá (27,0%) do que na zona oeste do Rio de Janeiro (18,15%). Cães machos, de pelo curto, mais pesados ou mantidos em ambiente externo apresentaram maiores chances de infecção. A tosse foi mais frequente em cães antígeno-positivos, sem associação com perda de peso ou letargia. Apesar das recomendações veterinárias, taxas substanciais persistem, sobretudo em comunidades de baixa renda, evidenciando falhas na governança preventiva. Esforços integrados de veterinários, autoridades e indústria são urgentemente necessários para aumentar a adesão à quimioprofilaxia e proteger a saúde canina em regiões endêmicas.

## Introduction

*Dirofilaria immitis* (Leidy 1856) Railliet & Henry, 1911, commonly known as heartworm, is a mosquito-borne nematode frequently found infecting dogs in Brazil. The reported prevalence in the country ranges from 2.1% to 62.2%, depending on the area studied (Bendas et al., 2017). An update on canine heartworm infection rates revealed that Brazilian coastal areas, once known for high rates, still posed a risk to unprotected dogs. The lowest rates in coastal areas occurred in the coolest locations within the temperate climate of southern Brazil, whereas the highest prevalence rates were observed in coastal tropical areas (Labarthe et al., 2014).

In low-income communities in the state of Rio de Janeiro, where dogs lack access to veterinary care, *D. immitis* infection rates are high. Dogs from these communities tested positive for *D. immitis* even in municipalities previously considered non-endemic (Araújo, 2024; Santos et al., 2021; Vieira et al., 2022). In the low-income western area of Rio de Janeiro city, 21.6% of dogs were antigen positive (Moraes-da-Silva et al., 2016) and in the low-income eastern area of the state (Maricá and São Pedro da Aldeia municipalities), 43.4% (23/53) of dogs were microfilaremic (Alberigi et al., 2023). These results highlight the role of unprotected dogs as a source of *D. immitis* microfilariae for mosquito vectors.

Prevalence reports including dogs with access to veterinary care tend to show lower infection rates, although they may still be high. A survey of 428 canine surplus samples obtained, regardless of suspicion of heartworm infection, from two private laboratories in the Rio de Janeiro Metropolitan Area revealed that 7% were antigen positive (Mendes-de-Almeida et al., 2021). A convenience survey conducted in Rio de Janeiro city sampled 131 dogs from small-animal practices and reported 14.5% antigen-positive results (Gouvêa de Almeida et al., 2023). A broader survey including 1,787 dogs with access to veterinary care from Rio de Janeiro (5% antigen positive) and Niterói (7.2% antigen positive) municipalities, among other areas of the state, revealed an overall 8.2% antigen-detected rate. In that study, of the 135 veterinary hospitals included, 108 (80%) routinely recommended heartworm preventives to their clients, revealing a gap between veterinarians' recommendations and owners' compliance. Clinical signs (cough, weight loss, lethargy or syncope) were significantly more common in heartworm-infected dogs according to questionnaire responses from dog owners (Guedes et al., 2024).

These results reinforce that canine *D. immitis* infections remain widely distributed in the Rio de Janeiro metropolitan area and in other municipalities of the state, particularly in coastal lowlands (Labarthe et al., 2014; Trancoso et al., 2020; Willi et al., 2017), despite veterinary recommendations for the use of heartworm preventives. Therefore, this study was conducted to survey canine *D. immitis* high-challenge areas in metropolitan Rio de Janeiro that continue to exhibit high infection rates despite the availability of veterinary care, chemoprophylaxis and educational materials.

## Material and Methods

### Study areas

Two areas with anecdotal reports of high canine *D. immitis* infection rates were studied. The western area of the Rio de Janeiro municipality, which includes a protected natural area known to favor mosquito vector breeding, was selected. The eastern lowland of Niterói and the western lowland of Maricá (Niterói-Maricá) are contiguous and were therefore analyzed as a single area although they are separated by a small natural reserve favorable to the breeding of *D. immitis* mosquito vectors. It is worth mentioning that the two areas (western Rio de Janeiro and Niterói-Maricá) are approximately 80km apart with the Guanabara Bay in between. Since the anecdotal reports of high canine *D. immitis* infection rates were progressively increasing in the area, they were elected to be surveyed.

### Animal inclusion and sampling

To be included, dogs had to be over 12 months of age and their owners had to formally agree with the study and declare that the animal had not received *D. immitis* chemoprophylaxis during the previous six months. All blood samples were obtained from the cephalic vein or from the jugular vein. The participant dogs were manually restrained using no muzzle, in the dogs’ households. The demographic, environmental and general health information, including the clinical signs cough, weight loss and lethargy, was recorded according to the owner’s information, using a standardized owner questionnaire. After inclusion, blood samples were collected in EDTA tubes, each identified by the dog's unique number and name. Whole blood samples were immediately tested for circulating *D. immitis* antigen using a point-of-care immunochromatographic test kit (Uranotest Dirofilaria®, Urano Vet SL, Barcelona, Spain), according to the manufacturer's instructions.

### Sample-size calculation

The canine population size was estimated according to World Health Organization recommendations, corresponding on average to 10% of the human population of the municipality (Bögel et al., 1990). Therefore, the canine population of each of the two areas was estimated on the basis of the reported human population (IBGE, 2010). To calculate the sample size for the western area of Rio de Janeiro, the canine population was estimated at 65,000 and the infection rate at 21.6% (Moraes-da-Silva et al., 2016). For Niterói-Maricá, the canine population was estimated at 12,746 and the *D. immitis* infection rate at 23.1% (Labarthe et al., 2014). The minimum sample size estimated for the western area of Rio de Janeiro was 259 dogs, and for Niterói-Maricá it was 267 dogs, following the formulas described by Cochran (1967) and Thrusfield (2005).

### Statistical analysis

Associations between each potential risk factor and *D. immitis* antigen detection were initially assessed using univariable logistic regression. Odds ratios (ORs) and their corresponding 95% confidence intervals (95% CIs) were calculated for each factor. The association between sex and body weight was assessed using Spearman's rank correlation coefficient. Associations between clinical signs and heartworm antigen detection were assessed using logistic regression models. Infection frequencies across clinical signs were compared using Pearson's chi-square or Fisher's exact tests, as appropriate. Statistical significance was set at p < 0.05. All analyses were performed using SPSS® (IBM®), version 29.0.

### Spatial analysis

Maps were generated in ArcGIS 10.1 using cartographic data from IBGE and MPERJ to depict hydrography, municipal boundaries and neighborhood divisions, and to geolocate canine blood-sampling points. Kernel interpolation indicated a relatively high concentration of sampling points near the coastline. Specific maps allowed the identification of the spatial distribution of *D. immitis* positive and negative results.

## Results

**Figure 1:**
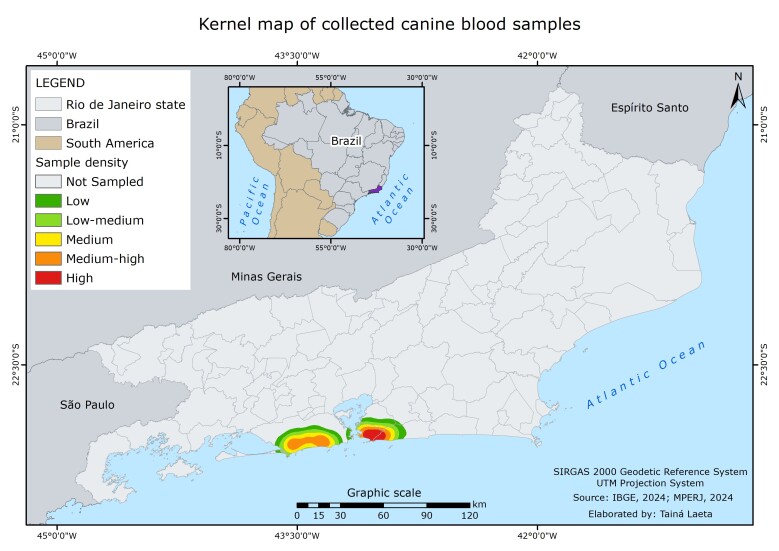
Map showing the spatial distribution of canine blood-sampling points in the municipalities of Rio de Janeiro, Niterói and Maricá, Rio de Janeiro state, Brazil.

**Figure 2:**
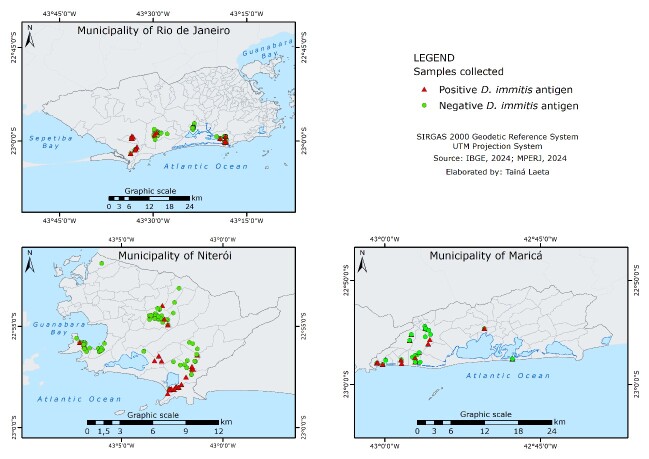
Maps of the municipalities of Rio de Janeiro and of Niterói and Maricá, Rio de Janeiro state, Brazil, showing *Dirofilaria immitis* antigen-detected canine samples (red triangles) and non-detected canine samples (green dots).

A total of 526 dogs from the two areas were included (Figure 1). The overall *D. immitis* antigen detection rate was 22.6% (119/526). The Niterói-Maricá area (27%; 72/267) was shown to have a higher infection rate than the western area of Rio de Janeiro (18.15%; 47/259) (χ^2^ = 5.8419; df = 1; p-value = 0.015649) (Figure 2).

Male dogs (OR 1.61; p < 0.05), dogs kept outdoors most of the time (OR 2.97; p < 0.0001) and short-coated dogs (OR 2.22; p < 0.01) were shown to be at greater risk. Heavier dogs (≥15 kg) were also at greater risk (OR for <15 kg = 0.53; p < 0.01). A correlation between sex and weight was not significant (r = 0.036; p = 0.45). Age, coat color and length of residence did not significantly influence heartworm antigen detection (Table 1).

**Table 1:** Potential risk factors for *Dirofilaria immitis* antigen positivity (HTW-Ag+) in dogs from the western area of the Rio de Janeiro municipality and from Niterói-Maricá, Rio de Janeiro, Brazil.

**Risk factor**	**N**	**HTW-Ag+ n (%)**	**OR (95% CI)**	**p value** *
**Total**	526	119 (22.6)	—	—
**Sex**				
Male	262	70 (26.7)	1.61 (1.06–2.45)	0.03
Female	264	49 (18.6)	Reference	—
**Age**				
≤4 yr	257	51 (19.8)	0.73 (0.48–1.10)	0.17
>4 yr	269	68 (25.3)	Reference	—
**Lifestyle**				
Mostly outdoors	261	83 (31.8)	2.97 (1.91–4.80)	<0.0001
Mostly indoors	265	36 (13.6)	Reference	—
**Fur length**				
Short	350	94 (26.8)	2.22 (1.36–3.60)	0.002
Medium-long	176	25 (14.2)	Reference	—
**Coat color**				
Black	175	37 (21.1)	0.89 (0.57–1.41)	0.72
Other colors	282	65 (23.0)	Reference	—
**Weight**				
<15 kg	319	58 (18.2)	0.53 (0.35–0.80)	0.003
≥15 kg	207	61 (29.5)	Reference	—
**Length of residence**				
<5 years	316	70 (22.1)	0.93 (0.62–1.42)	0.83
≥5 years	210	49 (23.3)	Reference	—

HTW-Ag+: *Dirofilaria immitis* antigen-positive result on the Uranotest Dirofilaria® (Urano Vet SL, Barcelona, Spain). OR: odds ratio; 95% CI: 95% confidence interval. N: number

* Univariate analysis.

According to the owners' reports, the most common clinical sign among antigen-positive dogs was cough (20.2%). Although cough may result from various respiratory conditions, such as kennel cough, upper respiratory infections or chronic bronchitis, fewer antigen-negative dogs coughed (11.8%; χ^2^ = 3.99; p < 0.05). No differences in weight loss or lethargy were observed between antigen-positive and antigen-negative dogs (Table 2).

**Table 2:** *Dirofilaria immitis* antigen detection in dogs living in the western area of Rio de Janeiro and in Niterói-Maricá, Rio de Janeiro, Brazil, according to clinical signs reported by their owners.

**HTW-Ag** 1	**Cough n/Total (%)**	**Weight loss n/Total (%)**	**Lethargy n/Total (%)**
Antigen-detected	24/119 (20.2)	14/119 (11.8)	20/119 (16.8)
No antigen-detected	48/407 (11.8)	26/407 (6.4)	59/407 (14.5)
**Total**	72/526 (13.7)	40/526 (7.6)	79/526 (15.0)
**χ^2^/p value**	3.99 / p=0.046	3.17 / p=0.075	0.28 / p=0.596

1 HTW-Ag: *Dirofilaria immitis* antigen result obtained with the Uranotest Dirofilaria® (Urano Vet SL, Barcelona, Spain).

## Discussion

The overall antigen-detection rate in the present survey was greater than 20%; therefore, the results for risk factors may be considered indicative of the likelihood of *D. immitis* infection, as observed previously (Labarthe et al., 2014). Diagnosis was pursued by rapid point-of-care testing because cross-reactions were not a concern in Rio de Janeiro: the parasites known to cross-react with *D. immitis* antigen tests, *Dirofilaria repens* and *Angiostrongylus vasorum*, are not endemic in the state. To our knowledge, there are no reports of *D. repens* infection in dogs in the state of Rio de Janeiro.

Dogs with access to veterinary care in the municipality of Rio de Janeiro had previously reported *D. immitis* infection rates of 7% (Mendes-de-Almeida et al., 2021) or 5% (Guedes et al., 2024). These rates are difficult to interpret, as all 43 veterinary businesses attending those dogs reported recommending heartworm chemoprophylaxis to their patients. The same was observed in Niterói, where the rate was 7.2% among dogs attending practices known to recommend chemoprophylaxis to their clients (Guedes et al., 2024). When the present survey tested dogs from areas including some low-income areas, the results were even more striking. In the two areas included, practices also reported routinely recommending chemoprophylaxis, but dogs received only minimal veterinary care; the percentage of antigen-positive dogs in both areas was much higher (22.6%). These results suggest that heartworm is poorly managed by stakeholders. When so many heartworm-positive dogs are kept close to dogs receiving veterinary care, with poor owner compliance or insufficiently persuasive veterinary recommendations, high infection rates can be expected, even among dogs regularly seen by veterinarians.

Chemoprophylactic products have been marketed in Brazil since 1992; many small-animal veterinary businesses operate in these municipalities; and canine heartworm disease has received strong educational attention from key opinion leaders. The high percentage of antigen-positive dogs kept by the low-income population surveyed may be a consequence of the owners’ vulnerability. This is an overall scenario evaluation once owner’s income range wasn’t evaluated nor could it be due to the Brazilian regulation that require special authorization. Even so, it should be considered that low-income communities’ owners often face competing priorities related to social vulnerability, and preventive pet care may not be perceived as a priority under these circumstances.

Considering that in the studied municipalities different income range communities are usually mingled and that their income wasn’t evaluated, the high percentage of antigen-positive dogs indicate low chemoprophylactic use by the populations. A Brazilian article has shown that dogs regularly seen by veterinarians are not properly protected (Guedes et al., 2024) reinforcing that poverty may contribute with lack of prophylaxis, but it is not the sole reason. Local veterinarians should stimulate proper prophylactic use and owners must be educated to understand the importance of prophylaxis. Therefore, in our view, closing the gap provoked by owners’ vulnerability requires coordinated action: local veterinarians offering accessible heartworm testing and risk-based counselling, the animal-health industry and authorities supporting chemoprophylaxis in high-challenge areas, and outreach campaigns adapted to the socioeconomic realities of the communities.

The risk factors that represent increased exposure to mosquito vectors (living mostly outdoors) and that facilitate mosquito access to canine skin and blood feeding (short fur) were shown to, at least, double the probability of infection, as previously reported for dogs receiving veterinary care (Guedes et al., 2024). Even though dogs living in areas with a lower prevalence (< 20%) may not show an increased odds ratio, veterinarians and pet owners must be aware that short-furred dogs left outside most of the time require year-round chemoprophylaxis. Veterinarians need to inform dog owners of, and reinforce, the seriousness of the risk, especially in tropical and humid areas.

Males and heavier dogs have been shown to be at slightly greater risk, possibly because of their greater production of olfactory cues (Takken & Verhulst, 2013), which may increase exposure outdoors (Selby et al., 1980). Although there has been no significant correlation between sex and weight, it must be considered that weight was evaluated according to weight range, which may have distorted the analyzes. Therefore, since males tend to be heavier than females, sex and weight represent similar potential risks, although with relatively low odds ratios. Nevertheless, exposure to mosquito-vectors outdoors enhances the chances of infection. Considering that larger dogs may spend more time outdoors and have greater home ranges increasing exposure to mosquito vectors while male dogs may roam more frequently in search of females, it can be speculated that males tend to be more exposed to the vectors (Monteiro et al., 2026).

White coat colour has previously been shown to have a protective effect (Labarthe et al., 2014), and light colours are less attractive to mosquitoes (Packard, 1903). On the other hand, black-coated dogs have been suggested to be more attractive to mosquitoes (Packard, 1903), but the present results did not confirm this as a risk factor for infection. The association between male sex and heavier body weight may also be partly explained by behavioural differences: larger dogs may spend more time outdoors and have greater home ranges, increasing exposure to mosquito vectors, while male dogs may roam more frequently in search of females, further increasing outdoor exposure time.

Different host-seeking behaviours of the endemic vectors known in the coastal lowlands of Rio de Janeiro state (*Ochlerotatus taeniorhynchus*, *Ochlerotatus scapularis* and *Culex quinquefasciatus*) (Labarthe et al., 1998; Lourenço-de-Oliveira & Deane, 1995), as well as the endemic potential vectors *Aedes aegypti* and *Aedes albopictus* (Serrão et al., 2001), may have influenced these results. Perceived colours may represent distinct stimuli for different species, especially given their circadian rhythms. Their habits are crepuscular, nocturnal or diurnal, and luminous intensities vary, thereby affecting species-specific colour preferences (Jung et al., 2021).

Therefore, it can be inferred that male, heavier, short-haired dogs that spend most of their time outdoors are at the greatest risk of *D. immitis* infection, as previously reported (Labarthe et al., 2014). This pattern suggests that the likelihood of infection increases under conditions that facilitate mosquito blood-feeding.

The only clinical sign that could have been suggestive to the attending veterinarian was cough. More antigen-positive than antigen-negative dogs coughed (χ^2^ = 3.99; p < 0.05), suggesting that all coughing dogs should be tested for heartworm infection. This is reinforced by the present results and by studies of dogs with access to veterinary care (Guedes et al., 2024), although one survey of 1,531 dogs did not find an association between clinical signs and antigen-positive status (Labarthe et al., 2014).

## Conclusions

The results confirm that *D. immitis* remains endemic in these areas of metropolitan Rio de Janeiro, with infection concentrated in male, short-coated, heavier, outdoor-living dogs, most of which remain clinically silent. This reinforces the need for veterinarians practicing in these areas to include heartworm testing in routine screening, regardless of the presence of clinical signs.

## Data Availability

The datasets generated and/or analysed during the current study are available from the corresponding author upon reasonable request.
